# Interfacial rheology for the assessment of potential health effects of inhaled carbon nanomaterials at variable breathing conditions

**DOI:** 10.1038/s41598-020-70909-y

**Published:** 2020-08-20

**Authors:** Dorota Kondej, Tomasz R. Sosnowski

**Affiliations:** 1 Central Institute for Labour Protection - National Research Institute, Czerniakowska 16, 00-701 Warsaw, Poland; 2 Faculty of Chemical and Process Engineering, Warsaw University of Technology, Waryńskiego 1, 00-645 Warsaw, Poland

**Keywords:** Methods of toxicology studies, Occupational toxicity, Carbon nanotubes and fullerenes, Chemical engineering, Biomedical engineering, Risk factors, Biosurfaces

## Abstract

Lung surface is the first line of contact between inhaled carbon nanomaterials, CNMs, and the organism, so this is the place where pulmonary health effects begin. The paper analyzes the influence of several CNMs (single- and multi-walled nanotubes with various surface area: 90–1,280 m^2^/g and aspect ratio: 8–3,750) on the surface-active properties of the lung surfactant, LS, model (Survanta). Effects of CNM concentration (0.1–1 mg/ml) and surface oscillation rate were determined using the oscillating drop method at simulated breathing conditions (2–10 s per cycle, 37 °C). Based on the values of apparent elasticity and viscosity of the interfacial region, new parameters: *S*_ε_ and *S*_μ_ were proposed to evaluate potential effect of particles on the LS at various breathing rates. Some of tested CNMs (e.g., COOH- functionalized short nanotubes) significantly influenced the surfactant dynamics, while the other had weaker effects even at high particle concentration. Analysis of changes in *S*_ε_ and *S*_μ_ provides a new way to evaluate of a possible disturbance of the basic functions of LS. The results show that the expected pulmonary effects caused by inhaled CNMs at variable breathing rate depend not only on particle concentration (inhaled dose) but also on their size, structure and surface properties.

## Introduction

Carbon nanomaterials, CNMs, are used (or can be formed as by-products) in many applications, which can be associated with an unintentional release of nanometric dust to the air^1–7^. The aerosol generated in this way is respirable (inhalable) and easily penetrates to the alveolar region of the respiratory system^8,9^. A substantial number of inhaled nanoparticles that are deposited in this region can promote direct interactions with the organism^10–12^. The first surface met by inhaled particles in the pulmonary region is a thin layer of alveolar liquid on the top of lung epithelium. This layer contains the mixture of specific compounds of the lung surfactant, LS – the structure, which plays a vital role in the physiological functions of the respiratory system^13^. It has been recognized that LS is sensitive to inhaled materials^9,14–16^, and the resulting impairment of LS composition and/or properties may contribute to serious health problems, including the acute lung injury and respiratory distress. Accordingly, the analysis of LS properties in the presence of external factors such as micro/nanoparticles or chemicals, can give a preliminary information regarding the possible respiratory health problems that may follow inhalation of these agents^17–20^.

Because of the high surface-to-volume ratio, inhaled nanoparticles present a particular threat for health even when their deposited mass is not high^21^. As shown by the recent studies^22,23^, effect of different nanomaterials on LS system may be highly specific and depend on several particle properties, such as the specific surface area, SSA or degree of hydrophobicity. Particle dose (concentration) is another essential factor in predicting lung toxicity^24^. Therefore, the current study is focused on the identification of minimal doses of CNMs with different properties that may cause direct disturbance of dynamic surface properties of LS.

The most important biophysical feature of LS is the ability to modulate surface tension during oscillatory variations of the alveolar interfacial area during breathing^25,26^. In the recent paper^22^, the oscillating pendant drop technique was applied to analyze interactions of nanocarbon particles with the model LS at 0.25 Hz as the typical rate of respiration. However, the question arises if the effect of nanoparticle inhalation will remain similar also at variable breathing pattern, which is a quite common situation in the real-life (people working or doing sport exercises, etc.). To answer that, in this paper we propose to investigate CNMs interactions with the model LS at dynamic conditions that correspond to different breathing rates (2–10 s per breath). We demonstrate the usefulness of the concepts of interfacial rheology in the determination of such effects, and we propose new parameters to assess them quantitatively.

### Quantitative analysis and the physiological role of LS dynamics

Several experimental systems have been used to investigate the dynamic surface-active properties of LS in vitro, including Langmuir trough, oscillating bubble tensiometer, constrained bubble/drop tensiometers^22,27,28^. However, not always appropriate conditions have been assured for such studies (e.g., physiological temperature and the surface area variations relevant to breathing cycle). Due to specific experimental conditions needed to determine dynamic properties of LS at the air/liquid interface, the quantitative analysis of the results also requires special measures. Up to now, the following numerical parameters have been most commonly used:

(a) the minimum value of the surface tension, σ_min_ (mN/m), recorded during periodical expansion–contraction cycles of simulated breathing.

(b) the amplitude of surface tension variations measured at such conditions (σ_max_ − σ_min_).

Clements et al.^29^ introduced the stability index, *SI,* that can be derived from the above-mentioned surface tension values:

1 $$SI= \frac{{\upsigma }_{\mathrm{max}}-{\upsigma }_{\mathrm{min}}}{0.5 \left({\upsigma }_{\mathrm{max}}+{\upsigma }_{\mathrm{min}}\right)}$$

Periodic variations of the surface tension during oscillations of the air/liquid interfacial area show a time-shift which result in the surface tension hysteresis. The normalized area of the hysteresis loop, *HA*_n_, was proposed by Notter et al.^30^ as the parameter to quantify this feature:

2 $${HA}_{n} ~ = ~\frac{{\left[ {\mathop \smallint \nolimits_{A} \upsigma {\text{d}}A~} \right]_{{{\text{expansion}}}} - \left[ {\mathop \smallint \nolimits_{A} \upsigma {\text{d}}A~} \right]_{{{\text{compression}}}} }}{{{A}_{{\max }} - {A}_{{\min }} }}$$

The hysteresis arises due to the time-dependent phenomena (relaxation) that may be attributed both to the intrinsic mechanical properties of the air/liquid interface and to the mass exchange of surface-active molecules between the surface layer and the underlying liquid. These dynamics may be described using 2D rheological formalism, i.e. by determining the apparent surface elasticity and the apparent surface viscosity^31,32^. Several authors studied the surface rheology of air/liquid interfaces with different LS models^33–35^, however they did not focus on exact relations to the real breathing conditions (in terms of temperature and surface deformation frequency). In the rheological formalism, the departure from the initial value of the surface tension: Δσ = σ − σ_0_ is connected with extensional deformation of the interface, γ:

3 $$\Delta\upsigma ={\Delta\upsigma }_{\mathrm{E}}+{\Delta\upsigma }_{\mathrm{V}}={\mathrm{ \varepsilon \gamma }}+\upmu {\dot{\upgamma}}$$

where $\dot{\upgamma }$ denotes the surface deformation rate. Equation (3) is the Kelvin–Voigt model of a visco-elastic air/liquid interface. The temporary surface dilatational deformation (extension) is defined as:

4 $$\upgamma =\frac{A-{A}_{0} }{{A}_{0}}$$

where *A* denotes the area of the interface at the given time instant *t*, and *A*_0_—the initial interfacial area (at *t* = 0). Rheological parameters of the interface are denoted as ε (N/m—dilatational surface elasticity) and μ (s N/m—dilatational surface viscosity), while Δσ_E_ and Δσ_L_ represent the elastic and viscous contribution of the surface tension deviation, respectively. Small-amplitude harmonic deformation of the interface with the angular frequency ω (rad/s) is described as:

5 $$\upgamma ={\upgamma }_{\mathrm{m}} \; \mathrm{cos\;\omega }t$$

and the corresponding visco-elastic response takes the form:

6 $$\Delta\upsigma ={\Delta\upsigma }_{\mathrm{m}} \; \mathrm{cos} \; \left(\upomega t+\mathrm{\varphi }\right)$$

where φ (rad) denotes the phase-shift (loss angle) that appears due to the viscous properties of the interfacial region. If φ > 0, the system shows the hysteresis. γ_m_ and Δσ_m_ in Eqs. (5) and (6) denote the amplitudes of surface deformation and surface tension, respectively. For purely elastic response of the interface, the loss angle φ is zero, and, if the viscosity predominates, φ approaches π/2. In general, the loss angle is equal:

7 $$\mathrm{\varphi }=\mathrm{arc\;tan}\frac{\mathrm{\omega \mu }}{\upvarepsilon }$$

which indicates that surface tension hysteresis depends both on the viscosity-to-elasticity ratio and on the frequency of surface deformation. The hysteresis is larger when viscosity dominates over elasticity, and when oscillations are slower (i.e., ω is larger).

For surfactants that undergo mass exchange (dynamic adsorption and desorption) with the air/liquid interface (as in LS system), the situation becomes more complicated since both ε and μ may be not constant. In such a case, these parameters depend on the current surface deformation γ(*t*) because the composition the interface is changing during surface dilation or contraction due to the mass exchange, i.e. surfactant diffusion and adsorption/desorption^31^. This process act towards the relaxation of Δσ. Obviously, surface deformation rate $\dot{\upgamma }$(*t*) also affects the amount of the surfactant transferred between the liquid and the interface at a given time period. Therefore, in the system considered in this work, both surface elasticity and surface viscosity should be considered as not the real (intrinsic) rheological characteristics of the interface but rather as the apparent parameters of the interfacial region. A more comprehensive discussion of these issues may be found elsewhere^31,33–37^.

The phenomenon of surface tension hysteresis has been analyzed and discussed in the relation to the physiological functions of LS^13,26,30,38–40^. It has been postulated that the hysteresis is related both to mechanical aspects of breathing (for instance, to the pressure–volume hysteresis in the lungs) and to the mass transfer phenomena on the pulmonary surface, including the hydrodynamic clearance of inhaled deposits from alveoli^26,38^. The rheological analysis of the interfacial dynamics discussed in the current work facilitates the quantitative assessment of these important processes.

## Results and discussion

**Figure 1:**
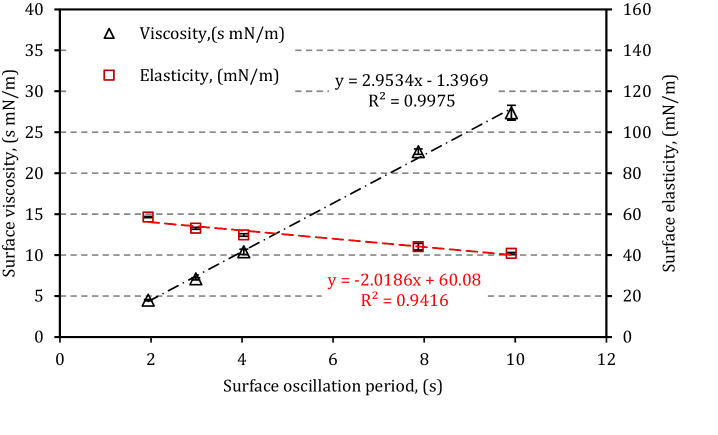
Surface dilatational elasticity, ε, and surface dilatational viscosity, µ, vs. oscillation period *T* of the interfacial area in LS model (Survanta^®^, 36.8 ± 0.2 °C). Error bars show the standard deviation (n = 3).

The first results obtained during drop oscillations of LS sample without particles at various frequencies revealed that both surface elasticity, ε, and viscosity, μ, depend linearly on the oscillation period (*T* = 2π/ω) in the studied surface deformation range, Fig. 1. These relationships can be expressed as:

8 $$\upvarepsilon \left(T\right)={S}_{\varepsilon }T+{I}_{\varepsilon }$$

9 $$\upmu \left(T\right)={S}_{\mu }T+{I}_{\mu }$$

where *S*_ε_ (N/ms) and *S*_µ_ (N/m) denote slopes of linear functions. They inform about the sensitivity of ε and μ to changes in the deformation rate corresponding to variable breathing frequency. Both parameters of interception, *I*_ε_ (N/m) and *I*_µ_ (s N/m), indicate the extrapolated values of surface elasticity or viscosity at *T* approaching 0, assuming that Eqs. (8) and (9) remain valid up to the infinite deformation frequency. *S*_ε_ and *S*_µ_ can be considered the sensitive numerical indicators of the dynamic surface tension-lowering activity of LS. As seen from Fig. 1, for pure LS (i.e. the control sample – no particles in the system) *S*_µ_ and *S*_ε_ are equal 2.95 mN/m and − 2.02 mN/m s, respectively. These numbers show that slower oscillations (i.e., higher *T* values) correspond to low surface elasticity and increased surface viscosity. Such findings fully agree with the theory since, in general, the viscosity/dissipation/relaxation are always prevailing at longer time-scale of deformation process^31,34^.

**Figure 2:**
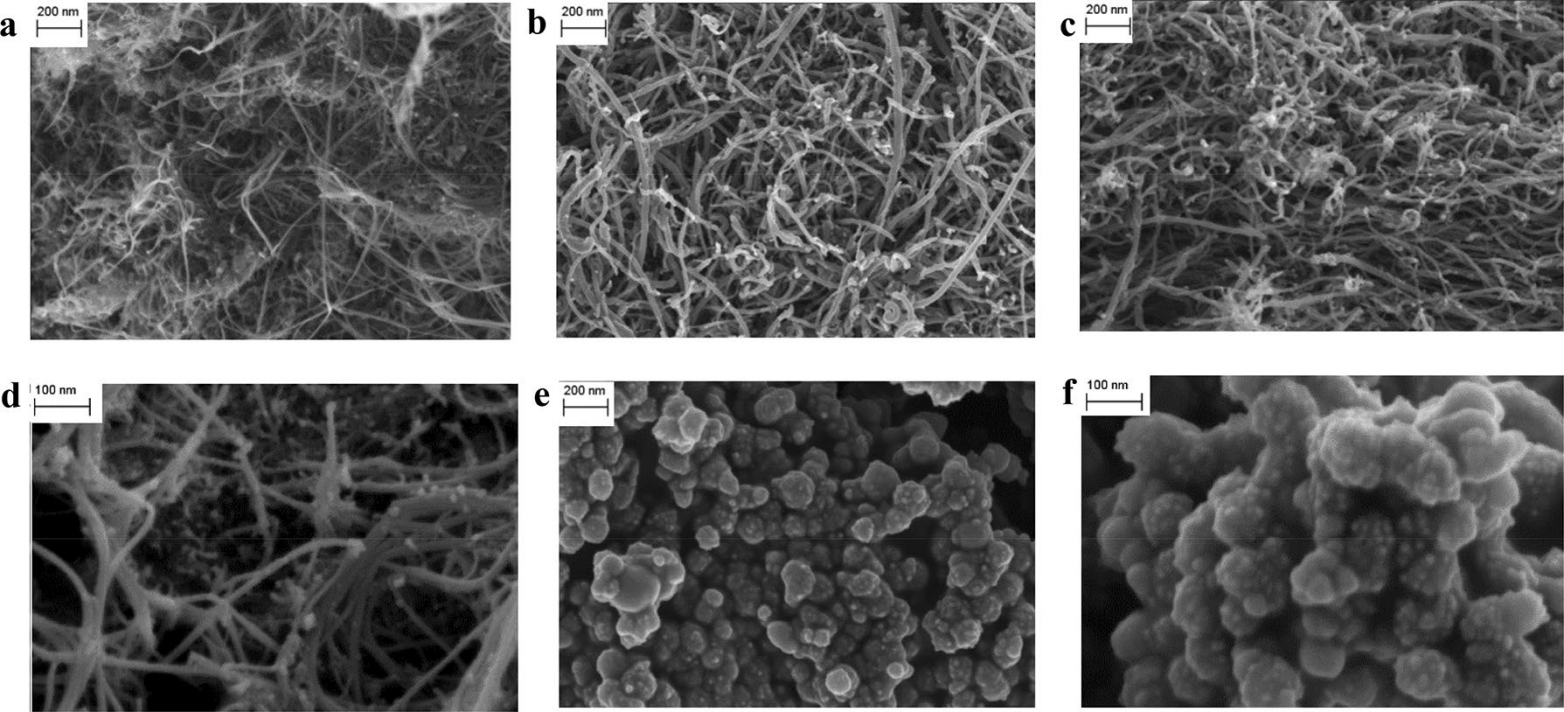
SEM micrographs of studied CNMs: **(a)** *MW-1*, **(b)** *MW-2*, **(c)** MW-f, **(d)** *SW*, **(e)** *NH*, **(f)** *NH-f*.

The morphology of studied CNMs is presented on SEM pictures (Fig. 2). Differences in the external diameter of nanotubes *MW-1*, *MW-2, MW-f* and *SW* are clearly seen (Fig. 2a–d, respectively) and agree with the data listed in Table 2. Both types of nanohorn particles *NH* and *NH-f* have similar morphology (note different scale in Fig. 2e,f), however they show various tendency to agglomerate. *NH* are agglomerated to a greater extent than *NH-f*, what results in the difference in SSA of these particles (Table 2).

**Figure 3:**
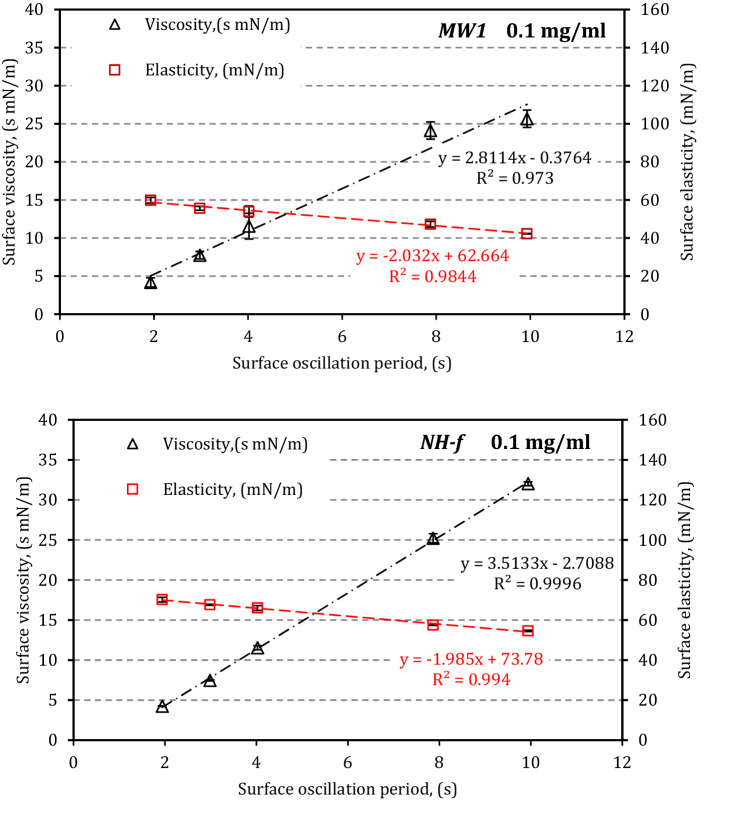
Examples of the linear relationships ε(*T*) and μ(*T*) (Eqs. 8 and 9) for LS in the presence of two types of CNMs (*MW-1* and *NH-f*) at particle concentration 0.1 mg/ml. Error bars show the standard deviation (n = 3).

CNMs added to LS cause a shift of both linear relationships ε(*T*) and μ(*T*)—Fig. 3. The relationships of surface elasticity and surface viscosity vs deformation period *T* are altered to a different extent, which clearly indicates a wide spectrum of effects induced by various nanoparticles as a function of concentration. Graph in Fig. 3 also confirms that it is possible to quantify the influence of CNMs on LS interfacial dynamics under variable breathing conditions by comparing the slopes *S*_ε_ and *S*_µ_.

**Figure 4:**
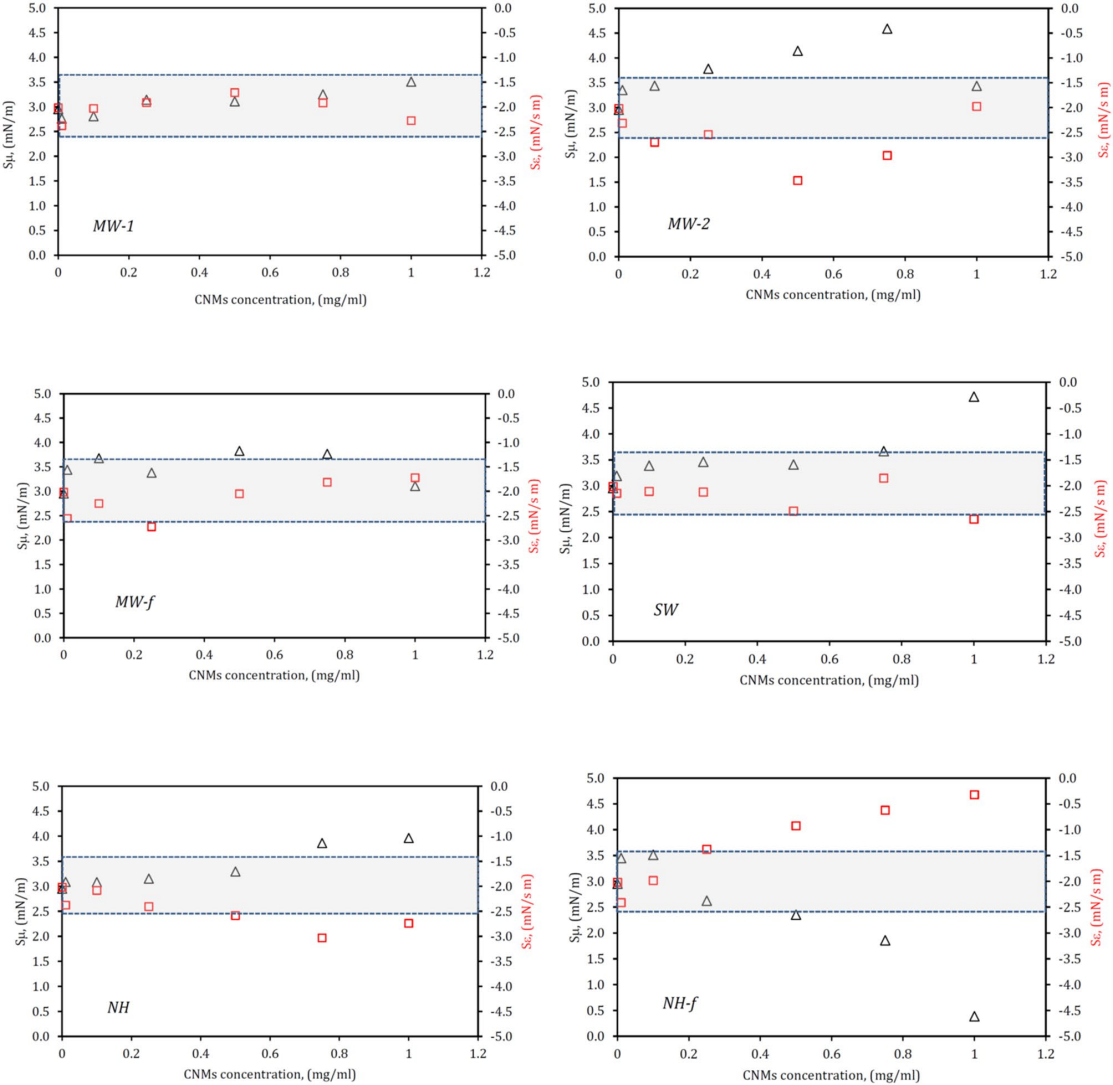
Relationships of *S*_µ_ (triangles—left axis) and *S*_ε_ (squares—right axis) vs. concentration of tested carbon nanoparticles in LS. Gray rectangle indicates the range of ± 25% deviation from the control values (LS without CNMs).

Values of *S*_ε_ and *S*_µ_ (determined for *T* = 2–10 s) vs concentration in the range of 0.1–1 mg/ml for all CNMs are plotted in Fig. 4. The window of quasi-constant *S*_ε_ and *S*_µ_ values assumed as ± 25% of these values for pure LS (Survanta), is also plotted in this graph (Fig. 3) to indicate clearly the concentration of a given CNM that corresponds to the most substantial alterations in LS dynamic surface activity. This relatively wide window embeds the scatter of data that is typically found in the multi-component system during the measurements of the dynamic surface tension during surface oscillations^32^. It is therefore assumed that results beyond this range may suggest that the dynamic surfactant activity has been altered to the degree that may have consequences for health. After analyzing of all presented data, the potential impact of tested carbon nanoparticles on the LS system is summarized in Table 1.

**Table 1:** Nanoparticle concentration range *c* (mg/ml) that corresponds to the strongest effects of CNMs on both parameters of the rheological response of LS surface: *S*_ε_ and *S*_μ_.

CNM type	Concentration range with the strongest effects on the slope of the surface elasticity, *S*_ε_(*T*)	Concentration range with the strongest effects on the slope of the surface viscosity, *S*_μ_(*T*)	Additional remarks
*MW-1*	–	–	–
*MW-2*	0.25 mg/ml < *c* < 0.75 mg/ml	0.25 mg/ml < *c* < 0.75 mg/ml	–
*MW-f*	–	–	–
*SW*	–	*c* > 0.75 mg/ml	–
*NH*	*c* > 0.5 mg/ml	*c* > 0.5 mg/ml	–
*NH-f*	*c* > 0.25 mg/ml	*c* > 0.5 mg/ml	The opposite trends of change in *S*_μ_(*T*) and *S*_ε_(*T*); *S*_μ_(*T*) ≈ 0 at 1 mg/ml

It is seen that not all tested CNMs induce direct changes in the dynamic activity of the surfactant, even at high nanoparticle concentration (~ 1 mg/ml). For instance, not unequivocal effects were found for multi-walled carbon nanotubes *MW-1* and *MW-f.* Although these nanoparticles are characterized by the similar length (10–30 μm), they differ both in the aspect ratio, AR (1,250–3,750 and 333–1,500, respectively) and the specific surface area, SSA (445 and 190 m^2^/g, respectively). This may suggest that these two parameters are not critical for the effects of carbon nanotubes on LS dynamics at the air/liquid interface. In contrast, data obtained for other CNMs suggest that geometrical features and SSA of nanoparticles are important. In addition, CNM concentration plays a big role in these effects.

Using the slopes *S*_ε_ and *S*_μ_ as indicators of the sensitivity of the apparent rheological parameters of the interface to variations in the oscillation (breathing) rate suggests that strong effects are caused by two types of nanoparticles of the relatively high SSA: *NH-f* (SSA ≈ 1,300 m^2^/g, AR = 8–25) and *SW* (SSA ≈ 950 m^2^/g, AR = 1,500) when their concentration exceeds 0.25–0.5 mg/ml. The analogous trend was found for *NH* particles at similar concentration, although these particles have much lower values of SSA and AR (≈ 400 m^2^/g, and ≤ 25, respectively). The observed effects may be caused by adsorption of LS molecules to the nanoparticles resulting in surfactant depletion, both on the interface and in the aqueous phase^22,41,42^. It should be noted though that the reported SSA values (Table 2) may be not equal to the area accessible for the adsorption of LS molecules. This issue will be discussed later.

**Table 2:** Carbon nanomaterials (CNMs) used in the studies: MWCNTs—multi-walled carbon nanotubes, SWCNTs—single-walled carbon nanotubes, CNHs—carbon nanohorns.

Particle designation in this study	Nanomaterials description and the supplier	External and internal particle diameter (*d*_*e*_ and *d*_*in*_)	Length (*L*)	Aspect ratio (AR = *L/d*_*e*_)	SSA (m^2^/g)
*MW-1*	MWCNTs 8 nm (Cheap Tubes Inc., USA)	< 8 nm/2–5 nm	10–30 μm	1,250–3,750	445 ± 10
*MW-2*	MWCNTs 50 nm (Cheap Tubes Inc., USA)	50–80 nm/5–10 nm	10–20 μm	125–400	92 ± 2
*MW-f*	COOH functionalized graphitized MWCNTs 20–30 nm (Cheap Tubes Inc., USA)	20–30 nm/5–10 nm	10–30 μm	333–1,500	190 ± 4
*SW*	SWCNTs (Sigma-Aldrich)	2 nm/ND	3 μm	1,500	935 ± 20
*NH*	CNHs, as grown (Sigma-Aldrich)	2–5 nm/ND	40–50 nm	8–25	396 ± 10
*NH-f*	CNHs, oxidized (Sigma-Aldrich)	2–5 nm/ND	40–50 nm	8–25	1,279 ± 28

*ND* no data, *SSA* specific surface area^22^.

Partly hydrophilic COOH-functionalized short nanotubes *NH-f* (nanohorns) with high SSA and low AR (~ 1,280 m^2^/g and 8–25, respectively), strongly modify dynamic interfacial properties of LS. In contrast to pure LS or the surfactant in the presence of other nanomaterials (where *S*_μ_ remains either almost constant or increases at high CNM concentration), a sharp decrease in *S*_μ_ is observed for *NH-f* particles at their concentrations above 0.5 mg/ml. It suggests a strong disturbance of LS dynamics at variable surface oscillation frequency. Reduction of *S*_μ_ to almost zero at high concentrations of *NH-f* particles means that changes in the frequency have no effect on the viscous response of LS interface. Accordingly, the surface tension hysteresis becomes independent on the breathing rate.

It is a completely opposite dynamic response of the air/liquid interface than in the normal (“healthy”) situation depicted in Fig. 1, where slower oscillations evidently increase the viscous character of the interface. Therefore, this effect of *NH-f* particles will decrease the σ-*A* hysteresis. As stated earlier, large hysteresis of the dynamic surface tension is essential for normal breathing mechanics and mass exchange in the lungs^13,38^, so the above findings should be considered negative for LS functions.

**Figure 5:**
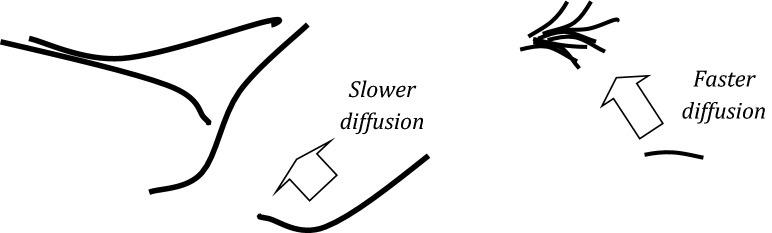
Schematic picture of different effects during aggregation of elongated nanotubes (slower diffusion and steric restrictions) and shorter nanocarbon particles (faster diffusion and better packing).

Another interesting observation is that *MW-2* nanotubes induce the maximum change in LS dynamics not at the highest (i.e. 1 mg/ml) but at a lower particle concentration (0.5–0.75 mg/ml). A similar effect has been already observed in the earlier studies^22^ and it can be attributed to the reduction of effective surface area of CNMs available for LS adsorption by particle aggregation favored by high particle concentration. This behavior of rod-like structures on the air/water interface and in the aqueous phase has been documented by other researchers^43,44^. It may be postulated that the assembling of nanoparticles in the liquid phase and on the air/liquid interface is also enhanced by low AR of particles thanks to reduction of steric restrictions and easier (faster) particle diffusion, Fig. 5. Such an assumption is partly supported by the data of Allegri et al.^45^ who were able to explain the suppression of the measured toxic effects by enhanced aggregation of short carbon nanotubes. This mechanism can also explain why the impact of *MW-2* (at 0.25–0.75 mg/ml concentration range) is stronger than the effect of *MW-1* particles, despite *MW-1* have significantly higher SSA.

Another explanation of these effects may be proposed after noting that SSA which are reported in Table 2 (in the Methods section) and considered in the ongoing discussion, were determined by nitrogen adsorption method. Such SSA values obviously overestimate the effective surface area of CNMs that is available for adsorption of large LS molecules. For instance, the inner surface of hydrophobic nanotubes with small internal diameter (such as *MW-1*) is poorly wetted by water, and this may considerably limit penetration of the liquid to the inner volume of *MW-1* nanotubes. Moreover, the access of relatively large surfactant molecules or their aggregates to these internal spaces is geometrically restricted. Therefore, the surfaces inside the thinnest nanotubes do not contribute to the mass-exchange and sorption processes that are decisive for the final effects of CNMs on LS dynamics at the air/liquid interface. It is also possible that different CNMs might be dispersed to a different degree when sonicated (see the Methods), which will also cause that their effective surface area is lower than declared. Inter-particle adhesion forces responsible for this effect depend on the CNM surface properties, dimensions and concentration, i.e. on the same factors which are important for aggregation.

## Methods

The CNMs studied in this work are characterized in Table 2. They have been carefully selected to cover a possibly broad range of dimensions, structure, specific surface area and surface composition.

The morphology of the particles was studied using SEM (Ultra Plus, Zeiss, Germany) after coating with Cr in Q150T ES sputter coater (Quorum Technologies, UK). The dynamic surface tension of a model lung surfactant (Survanta—Abbott Labs., France) was measured during sinusoidal oscillations of a pendant drop (PAT-1M drop shape tensiometer, Sinterface Technologies, Germany). Survanta is an animal-derived (bovine) mixture that contains 25 mg/ml phospholipids (including 11.0–15.5 mg/ml disaturated phosphatidylcholine), 0.5–1.75 mg/ml triglycerides, 1.4–3.5 mg/ml free fatty acids, and less than 1.0 mg/ml SP-B and SP-C protein) in sterile 0.9% sodium chloride solution. Mixtures of LS with each type of CNM were prepared by mixing Survanta with pre-sonicated suspensions of nanoparticles in ultra-pure water (ultrasonic energy: 4.8 kJ/ml). This allowed to obtain the mixtures with five CNM concentrations: *c* = 0.1, 0.25, 0.5, 0.75 and 1 mg/ml. The lowest concentrations correspond to the predicted values after human exposure to highly contaminated dusty environment^40^. The highest values (0.5–1 mg/ml) of nanoparticle concentrations are less realistic, however they allow to shed light on the mechanism of the physicochemical interactions which are less easily detected when CNMs concentration is low. The amount of Survanta and aqueous suspensions of CNMs were adjusted to obtain the same LS concentration in each final sample (2.5 mg/ml), identical to the LS concentration in the control sample without CNMs. The surface tension at the beginning of drop pulsation, i.e. after system equilibration (600 s) was in the range of 35–50 mN/m, depending on CNMs type and concentration. Droplets with the initial interfacial area of 16 mm^2^ were pulsated at physiological temperature 36.8 ± 0.2 °C at 20% oscillation amplitude (+ 10% and -10% of *A*_0_), applying five values of the oscillation frequency: 0.1, 0.125, 0.25, 0.33 and 0.5 Hz (the oscillation period, *T* = 2, 3, 4, 8 and 10 s).

## Conclusions

The basic physicochemical functions of the lung surfactant are related to the dynamic surface phenomena in the pulmonary alveoli during the breathing cycle. Presented in vitro results confirm the possibility of disturbance of these phenomena by carbon nanomaterials (short or long, single-walled or multi-walled nanotubes) that may be present in the LS system after inhalation at high exposures. The paper introduces new quantitative parameters which help to quantify such effects at conditions that correspond to variable breathing frequency which reflect different levels of physical activity of the body.

It was shown that the influence of CNMs on LS dynamics can be assessed by tracing the changes of the apparent surface rheological parameters of air/liquid interface at different rates of surface deformation. The CNMs studied here alter the surface activity of LS to a different degree under conditions that correspond to variable respiration frequencies. These changes are very specific, i.e. they depend not only on CNM concentration but also on other particle characteristics, and, in general, they are difficult to be predicted a priori. However, the measured phenomena are of high importance in the assessment of a possible impact of inhaled carbon nanomaterials on the respiratory functions in the real-life conditions where changes in the level of physical activity (i.e., in the breathing rate) are common.

The results show that various CNMs differently affect the LS dynamics, and that the effects depend in a distinctive way on the concentration of a given nanomaterial in the system. It has been confirmed that the specific surface area of carbon nanoparticles is one of the key factors since the interaction with LS are partly governed by adsorption of surfactant molecules to the nanomaterials. However, it was also shown that the nominal SSA values cannot be unequivocally correlated with the effect on LS. It is explained by differences in CNMs dispersion in the aqueous phase and by noting that SSA values measured by sorption of small molecules (nitrogen) overstate the CNMs surface area accessible for large surfactant molecules and their aggregates. The discussed effect was observed, among others, for multi-walled carbon nanotubes *SW*. It was also found that in some cases (i.e., *MW-2*) nanoparticles probably aggregate stronger above a certain concentration, and this phenomenon further reduces the particle surface area available for LS adsorption. The discussed effect is more important for nanoparticles with low aspect ratio, since they diffuse and aggregate more easily, and form more packed aggregated structures.

The most marked effect of CNMs on LS surface dynamics was observed in the case of functionalized (i.e. hydrophilic) carbon nanohorns with high SSA (*MW-f*). The presence of such nanoparticles in LS has a unique effect on the parameters *S*_ε_ and *S*_μ_ that have been introduced to trace the relationship between ε (surface elasticity) or μ (surface viscosity), and the time-scale of surface deformations, i.e. *T* (oscillation period). The viscous response of the model alveolar surface is completely suppressed by *MW-f* nanoparticles at high concentrations, so they reduce the surface tension hysteresis even at a slow deformation (breathing) rates. This suggests that LS will behave abnormally during the respiratory cycle, which may impair the essential pulmonary functions, including ventilation mechanics and the mass transfer.

The results not only indicate that inhaled carbon nanotubes and nanohorns with different surface properties may be responsible for health hazard due to specific physicochemical phenomena on the lung surface, but they also show that these effects may be dependent on respiratory dynamics that is related to different physical activity of the body. Such a dependency of interfacial effects in the LS system on the breathing rate provides a new information required for a more complete analysis of potential risks from inhaled carbon nanoparticles.

## Data Availability

Raw experimental data available on request.
